# Four Weeks of Time-Restricted Feeding Combined with Resistance Training Does Not Differentially Influence Measures of Body Composition, Muscle Performance, Resting Energy Expenditure, and Blood Biomarkers

**DOI:** 10.3390/nu12041126

**Published:** 2020-04-17

**Authors:** Matthew T. Stratton, Grant M. Tinsley, Michaela G. Alesi, Garrett M. Hester, Alex A. Olmos, Paul R. Serafini, Andrew S. Modjeski, Gerald T. Mangine, Kelsey King, Shelby N. Savage, Austin T. Webb, Trisha A. VanDusseldorp

**Affiliations:** 1 Department of Exercise Science and Sport Management, Kennesaw State University, Kennesaw, GA 30144, USA; matthew.stratton@ttu.edu (M.T.S.); malesi@students.kennesaw.edu (M.G.A.); ghester4@kennesaw.edu (G.M.H.); aolmos@highlands.edu (A.A.O.); pserafi1@students.kennesaw.edu (P.R.S.); modjeskia@gmail.com (A.S.M.); gmangine@kennesaw.edu (G.T.M.); kk12309@georgiasouthern.edu (K.K.); ssavage9@students.kennesaw.edu (S.N.S.); awebb37@students.kennesaw.edu (A.T.W.); 2 Department of Kinesiology and Sport Management, Texas Tech University, Lubbock, TX 79409, USA; grant.tinsley@ttu.edu; 3 Department of Kinesiology and Wellness, Georgia Highlands College, Cartersville, GA 30121, USA; 4 Department of Rehabilitation Sciences, Georgia Southern University Armstrong Campus, Savannah, GA 31419, USA

**Keywords:** intermittent fasting, time-restricted feeding, fasting, caloric restriction, body composition, resistance training

## Abstract

Recently, interest in time-restricted feeding (TRF) has increased from reports highlighting improvements in body composition and muscular performance measures. Twenty-six recreationally active males were randomly assigned to either TRF (*n* = 13; ~22.9 years; 82.0 kg; 178.1 cm; 8 h eating window, 25% caloric deficit, 1.8 g/kg/day protein) or normal diet (ND; *n* = 13; ~22.5 years; 83.3 kg; 177.5 cm; normal meal pattern; 25% caloric deficit, 1.8 g/kg/day protein) groups. Participants underwent 4-weeks of supervised full body resistance training. Changes in body composition (fat mass (FM), fat free mass (FFM), and body fat percentage (BF%)), skeletal muscle cross sectional area (CSA) and muscle thickness (MT) of the vastus lateralis (VL), rectus femoris, (RF), and biceps brachii (BB) muscles, resting energy expenditure (REE), muscular performance, blood biomarkers, and psychometric parameters were assessed. Significant (*p* < 0.05) decreases were noted in BM, FM, BF%, testosterone, adiponectin, and REE, along with significant increases in BP_1RM_, LP_1RM_, VJ_HT_, VJ_PP_, VL_CSA_, BB_CSA_, and BB_MT_ in both groups. Plasma cortisol levels were significantly elevated at post (*p* = 0.018) only in ND. Additionally, FFM was maintained equally between groups. Thus, a TRF style of eating does not enhance reductions in FM over caloric restriction alone during a 4-week hypocaloric diet.

## 1. Introduction

Fasting can be defined as abstinence from ingesting food and caloric beverages for specified time periods, ranging from hours to several weeks [1,2]. Recently, fasting has grown from its well-documented roots in religious practices to a common dietary strategy in both athletic and medical fields, often for the goal of improving body composition or cardiometabolic health markers [3,4]. Currently, there are a multitude of fasting protocols categorized as intermittent fasting (IF) including alternate day fasting (ADF) and modified alternate day fasting (mADF), whole day fasting, and time-restricted feeding (TRF). While a comprehensive examination of the differences between the various forms of IF is beyond the scope of this report (for reviews, see Tinsley et al., (2015b) [5] and Mattson et al., (2017) [6]), it is important to highlight some commonalities. Each variant utilizes a defined period of abstinence from all calories that extends the overnight fast and often leads to reductions in overall caloric intake [7,8], ranging from 25 to 38% [8,9,10]. This has led many to question whether changes in the physiological outcomes (e.g., body composition and cardiometabolic health markers) often associated with fasting protocols are attributed to the unique benefits of fasting itself, or solely due to the resultant caloric restriction. While previous investigations have examined the potential impact of IF on body composition [9,11,12,13,14,15,16] and metabolic health markers [9,13,14,17,18], the majority of investigations have been carried out in overweight and obese populations and without the implementation of a structured exercise program [12,13,14,15,16,17,19,20]. Furthermore, these investigations have primarily employed either ADF or mADF styles of fasting, as opposed to the more commonly practiced TRF.

TRF is traditionally characterized as daily caloric abstinence ranging from 12 to 20 h followed by ad libitum food and fluid consumption for the remaining hours of the 24-h period [5]. Unfortunately, there is a dearth of scientific literature on this form of IF in general and a particularly noticeable lack of data in exercising populations, among whom TRF is often practiced. Two common TRF variations involve a daily 16-h fast with an 8-h feeding window (16/8; e.g., the Lean Gains Diet) or a daily 20-h fast with 4-h feeding window (20/4; e.g., the Warrior Diet) [5]. To date, only three previous interventions have been carried out in resistance-trained populations, two of which employed the 16/8 protocol [21,22], while the other examined a modified 20/4 regimen [23]. First, the work by Tinsley et al. (2016) [23] examined fasting on alternating days. Specifically, previously untrained men were asked to follow a fasting protocol for 8-weeks consisting of 4-h feeding windows four days per week, with ad libitum feeding the remaining three days per week, on which they also resistance trained. Despite participants in the TRF group reporting consumption of, on average, 667 kcals, 75 g of carbohydrates, 25 g of fat, and 30 g of protein (equating to 0.4 g/kg) less on fasting days than non-fasting days, no significant differences were noted for body mass (BM), lean soft tissue (LST), or fat mass (FM). Furthermore, muscular performance adaptations were not inhibited in the TRF group, with improvements noted for both lower body strength and endurance as well as upper body strength at the end of the 8-week investigation.

Similar findings were reported by Moro and colleagues [21], who investigated a 16/8 TRF protocol in well-trained men. Unlike Tinsley et al. (2016), participants in the TRF group were required to fast everyday throughout the duration of the study instead of just on non-training days. Furthermore, both groups reported consuming roughly the same number of kcals (~3000 kcals/day) and protein (~2.0g/kg/day) spread out across three meals. The non-fasting group consumed their meals at 8 am, 1 pm, and 8 pm, while the TRF group consumed their meals at 1 pm, 4 pm, and 8 pm. Subsequently, all participants underwent supervised training conducted thrice weekly, consisting of heavy compound movements to failure. Body composition, resting metabolic rate, basal levels of anabolic and hunger hormones, and muscular performance were examined pre- and post- the 8-week intervention. The TRF group experienced significant decreases in FM, leptin, total testosterone, adiponectin, IGF-1, and increases in the adipokine adiponectin. Although, it must be noted that significant differences for leptin were no longer apparent once corrected for BM. Furthermore, there were no significant differences found in fat free mass (FFM) or muscular performance. Finally, a similar investigation was carried out in resistance trained females by Tinsley and colleagues in 2019 [22]. Twenty-four participants underwent 8-weeks of supervised training while, much like Moro et al. (2016), following either a 16/8 fasting protocol or eating regularly from breakfast until the end of day. Furthermore, all participants consumed an average of ~1.6g/kg/day of protein. In a per protocol analysis of 24 participants who completed the intervention and complied with study procedures, a significant group by time interaction indicated greater loss of FM in the TRF and TRF plus beta-hydroxy-beta-methylbutyrate (HMB) supplementation groups relative to the normal diet group at the 4-week timepoint, but the FM loss only remained statistically significant for the TRF plus HMB group by the end of the study. However, no significant differences between groups were observed in the intention-to-treat analysis that included all 40 participants who entered the study. Once again, no difference in changes in FFM were noted between groups, with all groups demonstrating an increase. Similarly, all groups displayed increased muscle thickness of the elbow flexors and knee extensors as well as muscular performance improvements without differences between groups. With the disparate findings in FM changes between the three previous studies, and the substantial interest in TRF programs to alter body composition, further investigation is required, employing a more robust method of body composition assessment (e.g., multi-compartment models compared to dual-energy X-ray absorptiometry [24]). Furthermore, due to the limited literature in resistance training populations, there is a need for further exploration of whether TRF influences adaptations to resistance training when calorie and protein intakes are controlled. Thus, the current investigation sought to expand on the work of Tinsley et al. [22,23] and Moro et al. [21] by further exploring the impact of short-term TRF. More specifically, Tinsley et al. 2019 [22] noted significant improvements in body composition at four weeks in the TRF group, but these changes no longer remained at eight weeks. Thus, four weeks was chosen for our investigation in order to further elucidate the potential effects of shorter time periods of calorie restrictive dieting. Additionally, 4-week caloric restriction time periods may also be more representative of field implementations of TRF in recreational populations (e.g., improvements in body composition or weight while peaking for strength sports). Furthermore, due to the limited research on resistance training and TRF, this data could not only be telling of the more acute changes in body composition, muscular performance, resting energy expenditure, and blood biomarkers, but be built upon by future research studies that implement a similar design for longer time periods (e.g., 8–24 weeks). Therefore, our objective was to investigate the effects of 16/8 TRF versus a normal meal distribution with equivalent calorie deficits and protein intake in both groups on measures of body composition, muscular performance, resting energy expenditure, and blood biomarkers following 4-weeks of resistance training in recreationally active men.

## 2. Materials and Methods

### 2.1. Experimental Design

**Figure 1:**
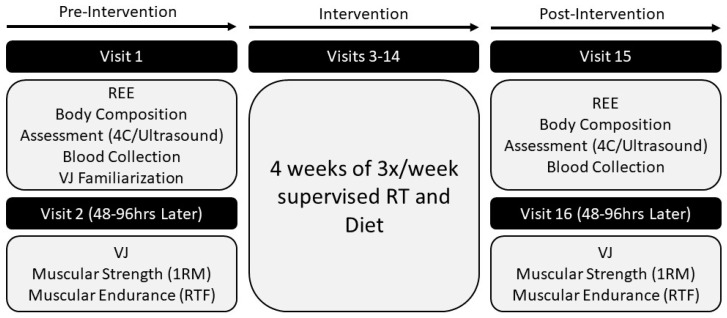
Study design overview. REE = resting energy expenditure; VJ = vertical jump; 4C = 4 compartment model of body composition; 1RM = 1 repetition maximum; RTF = repetitions until failure; RT = resistance training.

The present investigation included a total of 16 visits (two pre-intervention laboratory visits, 12 training visits, two post-intervention laboratory visits) over a 6-week period. Prior to the commencement of testing, participants were informed of the requirements of participating in the study, reviewed and signed an Institutional Review Board (IRB)-approved consent form indicating their willingness to participate, and completed health history and risk stratification questionnaires. Participants were then randomly assigned (i.e., simple randomization) to one of two groups: TRF with a 25% caloric deficit or normal daily feeding (ND) with a 25% caloric deficit. All laboratory testing was conducted in the Exercise Physiology Laboratory, while all supervised training sessions were held at the on-site resistance training facility. The study was approved by the Kennesaw State University (KSU) IRB (study #19-014). All data were collected in accordance with the Declaration of Helsinki. An outline of the study timeline is displayed in Figure 1.

### 2.2. Participants

The desired number of participants for a two-way repeated measures analysis of variance was determined via a power analysis (G*Power v3.0.10, Universitat Dusseldorf, Germany) utilizing an effect size of 0.3 and a power of 0.8 (generic input). It was determined that a total sample size of 24 would be needed via the power analysis. Thirty-two males between the ages of 18 and 35 years were recruited for the study to account for potential drop-outs or participants who did not adhere to the investigation guidelines. All participants were categorized as recreationally active, which was operationally defined as engaging in resistance training 2–4 times per week for the past six months. Participants were free of orthopedic injuries (within the past five years) as well as cardiovascular, metabolic, and pulmonary disorders that would contraindicate participation in the testing or training protocols involved in the study. All participants had not undergone any significant weight loss (≥10% of body weight) in the six months prior to enrollment and were not currently practicing any TRF protocols. Individuals who had a history of eating disorders were excluded from participation. Additionally, any participant using anabolic steroids or currently taking medications (e.g., steroidal and non-steroidal) or dietary supplements (e.g., creatine, beta-alanine, fish oil) that could have interfered with the study results were not enrolled.

### 2.3. Laboratory Assessments

#### 2.3.1. Overview

During the initial visit (Visit 1), participants were asked to arrive in athletic clothing after an overnight fast (≥8 h) and abstention from caffeine (≥12 h) and strenuous exercise (≥24 h). Participants were given an overview of the study, provided their written consent to participate, and completed all medical, exercise, and dietary questionnaires. All volunteers were then randomized into one of two groups, TRF or ND, and completed an assessment of resting energy expenditure (REE) via indirect calorimetry. Following these procedures, participants underwent a blood draw for the collection of serum and plasma, body composition assessment via the 4-compartment (4C) model, ultrasonography for assessment of muscle morphology, and vertical jump (VJ) familiarization.

Visit 2 was completed 48–96 h following Visit 1 and consisted of measures of exercise performance including VJ, leg press one-repetition maximum (LP*_1RM_*), bench press one-repetition maximum (BP*_1RM_*), leg press repetitions to failure (LP*_RTF_*) with a load corresponding to 65% of LP*_1RM_*, and bench press repetitions to failure (BP*_RTF_*) with a load corresponding to 65% of BP*_1RM_*. Upon completion of all baseline data collection, participants underwent four consecutive weeks of a periodized resistance training program consisting of 3x weekly full body routines in a daily undulating periodization scheme with both pre-intervention visits repeated following training completion. Each participant’s post-testing visit time occurred within ±2 h of the pre-testing visit time. Visit 15 occurred 72–96 h after the final training session. Visit 16 was the final visit, occurring 48–96 h after Visit 15.

#### 2.3.2. Anthropometric Measurements

Body mass was measured at the beginning of each body composition trial using a calibrated scale (COSMED USA, Inc., Concord, CA, USA). Participants wore only compression shorts and a swim cap during body mass assessments. Height was measured following enrollment into the investigation during the first visit. All height measurements were completed using the same calibrated stadiometer (Tanita WB 3000, Arlington Heights, IL, USA).

#### 2.3.3. Body Composition

During pre- and post-intervention assessments, a 4C model was utilized to assess body composition. This model necessitated assessments via dual energy X-ray absorptiometry (DXA; Lunar iDXA, General Electric, Chicago, IL, USA), air displacement plethysmography (ADP; BodPod, COSMED USA, Inc., Concord, CA, USA), and bioelectrical impedance analysis (BIA; InBody 770, InBody, Cerritos, CA, USA). All pieces of equipment were calibrated the morning of each assessment in accordance with the manufacturers guidelines. DXA, ADP, and BIA provided estimates of bone mineral content, total body volume, and total body water, respectively. These variables were entered into the 4C equation of Wang et al. [25] to determine FM, FFM, and body fat percentage (BF%) for each participant.

#### 2.3.4. Ultrasound

Muscle morphology (cross-sectional area (CSA), muscle thickness (MT), and muscle quality (echo intensity (EI)) was evaluated using ultrasound (General Electric LOGIQ P7, Wauwatosa, WI) with a 12-MHz linear probe scanning head for the following musculature: *m. rectus femoris* (RF) [26], *m. vastus lateralis* (VL) [27], and *m. biceps brachii* (BB) [28]. All images were taken by the same technician, who had previously demonstrated “excellent” reliability in a population of college-aged males and females (*n* = 10; RF: CSA: intraclass correlation (ICC) = 0.98, standard error of the mean (SEM) = 0.350, coefficient of variation (CV) = 2.67%; MT: ICC = 0.96, SEM = 0.059, CV = 2.59%; VL: CSA: ICC = 0.99, SEM = 0.201, CV = 0.61%; MT: ICC = 0.97, SEM = 0.079, CV = 3.70%; BB: CSA: ICC = 0.99, SEM = 0.085, CV = 0.97%; MT: ICC = 0.99, SEM = 0.068, CV = 3.37%). Preceding all assessments, a standard depth and gain was set. To ensure that the probe moved along a transverse plane of the muscle, foam padding was taped to the skin perpendicular to the longitudinal axis of the desired site. Three images were collected at each site. Subcutaneous fat thickness for both muscles was measured using the straight line function and used to calculate normalized EI, as suggested by Young et al. (2015) [29]. The average of the closest two images were subsequently used for analysis. All images were transferred to a personal computer for analysis via ImageJ (National Institutes of Health, Bethesda, MD, USA, version 1.45s). CSA was determined using the polygon function in ImageJ to select as much of the target muscle as possible without any surrounding bone or fascia. The distance between the superficial aponeurosis to the deep aponeurosis was used to determine muscle thickness. Muscle quality was determined from the echo intensity values assessed from the same region as CSA. The mean echo intensity value was calculated as a grey scale value between 0 (black) and 255 (white) arbitrary units. Specific sites for ultrasound were collected as previously described: (1) RF at 50% of the longitudinal distance of the anterior superior iliac spine and the superior border of the patella (5 cm depth; 50 gain) [30]; (2) VL at 50% of the longitudinal distance of the greater trochanter to the lateral epicondyle of the femur while maintaining a 10° knee angle (5 cm depth; 50 gain) [31]; and (3) BB at 66% of the distance from the medial acromion process to the cubital fossa while lying in a supine position with shoulder abducted (4 cm depth; 58 gain) [28].

#### 2.3.5. Resting Energy Expenditure

REE was assessed via indirect calorimetry (Parvo Medics TrueOne 2400, Parvo Medics Inc., Salt Lake City, UT, USA). Gas and flow calibrations were performed each morning according to the manufacturer’s specifications, and the pre-assessment procedures of Compher et al. [32] were utilized. Volunteers laid supine in a dark room for 20–30 min with a ventilated hood placed over their face and neck. REE was determined based on a 5-min interval of measured volume of oxygen consumption (VO_2_) with a coefficient of variation (CV) ≤5%.

#### 2.3.6. Blood Sampling and Analysis

The participant’s fasted blood samples were obtained pre- and post-intervention. Blood samples were obtained from an antecubital vein by a research team member who was trained in phlebotomy. Approximately 20 mL of blood was drawn into serum-separating- (SST) (for serum collection) and ethylenediaminetetraacetic acid (EDTA)-treated Vacutainer^®^ tubes (for plasma). SST tubes were allowed to clot for 10 min prior to centrifugation, while EDTA treated tubes were gently inverted and centrifuged. Samples were centrifuged for 10 min at 3600 rpm at 4 °C. The resulting serum and plasma were aliquoted and stored at –80 °C until analysis.

Plasma cortisol (EIAHCOR, Invitrogen, CA, USA), serum testosterone (ab108666, Abcam, CA, USA), serum adiponectin (KHP0041, Invitrogen, CA, USA), leptin (KAC2281, Invitrogen, CA, USA), and total ghrelin (BMS2192, Invitrogen, CA, USA) were all analyzed in duplicate via enzyme-linked immunosorbent assays (ELISA). All ELISA kits were read using a FilterMax F5 Multi-Mode Microplate Reader (Molecular Devices; San Jose, CA, USA), according to the kit guidelines. The inter-assay coefficient of variations (CV) and values for all biomarkers can be found in Table 4.

#### 2.3.7. Vertical Jump

Vertical jump height (VJ*_HT_*) and peak power (VJ*_PP_*) data were collected using a portable force plate sampling at 1000 Hz (AccuPower Solutions, Watertown, MA, USA). Three maximal counter movement jumps (CMJ) were performed with one minute of rest between attempts. All CMJ were carried out with the participants placing their hands on their hips and rapidly descending to a self-selected depth prior to jumping with maximal effort. The participants were instructed to avoid performing a tuck jump. VJ*_HT_* and VJ*_PP_* were all assessed during the concentric phase of the CMJ. Data were analyzed via AccuPower software (Version 2.0; AccuPower Solutions, Watertown, MA, USA).

#### 2.3.8. Estimated One Repetition Maximum

Estimated one repetition maximums (e1RM) were determined for the barbell bench press and leg press exercises. For both exercises, participants were asked to complete a specific warm-up, as previously described [33], consisting of five repetitions with 20% of the estimated 1RM, followed by three repetitions at 50% of the estimated 1RM, two repetitions at 75% 1RM, and finally one repetition at 85% of their estimated 1RM. Subsequently, sets of five repetitions were completed with increasing weight until the participant could no longer complete the set, with 5 min of rest between maximal attempts. All maximal attempt weights were selected by the investigator based on the repetitions in reserve reported by the participant at the completion of each set and visual bar speed throughout each attempt. Each participant’s sub 5RM was determined within three to five attempts. Once each participant’s sub 5RM was achieved, results were entered into the Brzycki 1RM estimation formula (weight lifted/[1.0278 – (0.0278 × repetitions performed)]) for the final determination of e1RM. The bench press was performed with the participant’s preferred grip width and the requirement that the bar made contact with the chest and returned to the starting position. A repetition was only considered ‘good’ (i.e., counted) if the participant completed the repetition with feet on the floor and hips and upper back remaining in contact with the bench. For a leg press attempt to be considered successful, the participant’s legs were required to descend to a 90° angle between the lower leg and thigh, then return to the starting position while the rest of the body remained in contact with the seat. Prior to all leg press sets, foot position was measured to ensure all maximal attempts were completed in the same manner. All sets were completed with trained spotters from the research team to assure proper technique and safety of all participants. 

#### 2.3.9. Muscular Endurance

Five minutes after leg press and bench press e1RM had been established, muscular endurance was determined utilizing 65% e1RM for a single set until muscular failure for both exercises. The order of exercise performed was the same as 1RM testing (i.e., bench press followed by leg press). Failure was defined as the first repetition that required assistance from a spotter, or in which a breakdown in technique was observed during the repetition. All attempts were performed with the same hand and foot placement as the 1RM testing and under the supervision of members of the research team to assure the safety of the participant. 

#### 2.3.10. Questionnaires

The perceived recovery scale (PRS) [34] was completed upon arrival to each training session, while the daily analyses of life demands for athletes (DALDA) [35] and visual analogue scales (VAS) for energy, desire to eat, hunger, fullness, and motivation to do physical tasks were completed upon arrival to the first training session of each week throughout the investigation. Furthermore, the DALDA and three-factor eating questionnaire (TFEQ-R18) [36] were completed during pre- and post-testing body composition visits, prior to ultrasound examination. The PRS was assessed on a 0–10 scale with 0 representing ‘very poorly recovered/extremely tired’ and 10 representing ‘very well recovered/highly energetic’. The VAS were also assessed on a scale ranging from 0 to 10 (0 = no energy, no desire to eat, no hunger, not full, no motivation and 10 = extremely energetic, extreme desire to eat, extremely hungry, extremely full, extremely motivated). The DALDA consists of two parts (A and B) and contains a combined total of 34 questions judged on a 3-point Likert-Scale (a = worse than normal, b = normal, c = better than normal). The TFEQ-R18 contains 17 questions based on a 4-point Likert-Scale (1 = definitely false, 2 = mostly false, 3 = mostly true, 4 = definitely true) and one question scored between 1–8 on eating restraint (1 = no restraint when eating, 8 = extreme restraint when eating). The TFEQ-R18 then provides scores for cognitive restraint, emotional eating, and uncontrolled eating between 0–100.

### 2.4. Intervention

#### 2.4.1. Dietary Program

Randomization into one of two groups occurred: (1) TRF: time-restricted feeding with all calorie and macronutrient consumption occurring within an 8 h period each day and a prescribed 25% caloric deficit; and (2) ND: normal daily feeding schedule with a prescribed 25% caloric deficit.

In addition to the 25% caloric restriction prescribed to both groups, all participants were asked to consume 1.8 g/kg/day of dietary protein in order to promote optimal FFM adaptations [37]. Additionally, on training days 50g of hydrolyzed whey protein isolate (ISO-100; Dymatize Enterprises LLC) was given to all participants upon the completion of each workout to ensure optimal post workout nutrient timing. The remaining calories were dispersed between carbohydrate and fat intake at the discretion of the participant. Maintenance calories were estimated as REE multiplied by a standard activity factor of 1.5. Therefore, the final equation for prescribed daily calories was as follows: (REE × 1.5) × 0.75. Participants in the TRF group were asked to consume all their required calories and macronutrients in an 8-h feeding window of either noon to 8 pm or 1 pm to 9 pm. Each participant selected their preferred eating window at study commencement and were required to utilize the same window throughout the study. The ND group had no time-based restrictions for their eating program. For the duration of the intervention, participants reported dietary intake for three days each week (two weekdays, one weekend day) using the MyFitnessPal application. A member of the research team checked food log compliance each week during resistance training sessions. Total calories, relative calories, carbohydrate (grams), fat (grams), and protein (grams) were collected from food logs. Average macronutrient and calorie intakes were compared between groups. 

#### 2.4.2. Resistance Training Protocol

**Figure 2:**
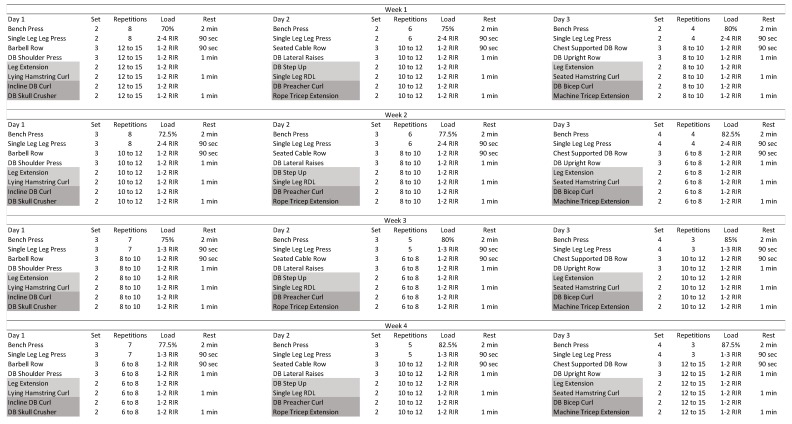
4-week resistance training program; All percentages were based off estimated 1RM at pretesting. RIR = Repetitions in Reserve; DB = Dumbbell; RDL = Romanian Deadlift; Sec = Seconds; Min = Minutes.

The 4-week training protocol consisted of full body sessions performed three times per week. The leg press and bench press were performed in all sessions, followed by a horizontal rowing exercise, a shoulder exercise, quadricep and hamstring dominant exercises performed in supersetted fashion, and tricep and bicep exercises performed as a superset. The bench press exercise was performed with two minutes of rest between sets, whereas the rest interval between all single leg press and horizontal rowing exercises was 90 s. Finally, all shoulder and supersetted exercises were separated by one minute of rest. The workouts proceeded in a daily undulating periodization pattern with bench and leg press repetitions ranging from 3 to 8. The bench press weights were based on percentages from the pre-intervention e1RM. All subsequent exercises were performed to a prescribed range of repetitions-in-reserve [33]. All training sessions were conducted under researcher supervision. Participants were required to check in at the beginning of all training sessions and complete the session as prescribed in order to be considered compliant with the training protocol. Training sessions occurred in the afternoon between 3 pm and 8 pm, during the participants’ feeding window. Participants who had less than a 90% compliance rate were excluded from the analysis. The resistance training program is displayed in Figure 2. 

### 2.5. Statistical Analysis

The assumption of normality was confirmed with the Shapiro–Wilk test (*p* > 0.05). Independent samples *t*-test were used to assess baseline differences between groups. Dependent variables were analyzed using two-way (group × time) repeated measures analyses of variance (ANOVA). In the event of a significant interaction, the POST timepoint was compared between groups using an independent samples *t*-test with a Bonferroni adjustment. Partial eta squared (η_p_^2^) effect sizes were calculated for analyses of variance findings, whereas Cohen’s *d* effect sizes were determined for the *t*-test results. A criterion alpha level of *p* ≤ 0.05 was used to determine statistical significance. Data were analyzed using the statistical package SPSS (Version 25.0, Chicago, IL, USA). All data were presented as mean ± SD (unless otherwise noted).

## 3. Results

### 3.1. Participants

Thirty-two participants were recruited to participate in the investigation, while 30 participants completed the study. Of the two participants who failed to complete the study, one voluntarily removed himself for personal reasons, while the second failed to adhere to post-testing timing procedures. Both of these participants were in the ND group. From the 30 participants that completed the study, 26 were included in the final analysis. One individual was excluded for lack of adherence to the fasting protocol (TRF group), and three were excluded (TRF: 1, ND: 2) for lack of dietary adherence (i.e., noncompliance with assigned macronutrient or kcal consumption) via dietary logs collected throughout the study. The independent samples *t*-tests demonstrated no significant differences between groups for baseline values of all dependent variables (Table 1). Participant characteristics for the final 26 participants included in the analysis are displayed in Table 1.

### 3.2. Dietary Intake

No significant differences were noted between groups for intakes of 4-week average protein, carbohydrate, fat, total calories, or calories per kilogram of bodyweight throughout the investigation (Table 2).

### 3.3. Body Composition and Muscle Morphology

Main effects for time where seen for BM (*p* = 0.001; η_p_^2^ = 0.355; TRF pre: 82.0 ± 10.6 kg, TRF post: 80.8 ± 10.9 kg; ND pre: 83.3 ± 14.9 kg, ND post: 81.9 ± 14.8 kg), FM (*p* < 0.001; η_p_^2^ = 0.806; TRF pre: 16.8 ± 8.6 kg, TRF post: 15.3 ± 8.5 kg; ND pre: 16.3 ± 8.5 kg, ND post: 14.9 ± 8.1 kg), and BF% (*p* < 0.001; η_p_^2^ = 0.834; TRF pre: 19.9 ± 8.3%, TRF post: 18.3 ± 8.2%; ND pre: 18.9 ± 7.4%, ND post 17.4 ± 7.4%). No group × time interactions were noted for BM, FM, FFM, or BF%.

No group × time interactions were found for the VL, RF, or BB measures (CSA, MT, EI); however, main effects for time were noted for VL CSA and VL EI as well as BB CSA and BB MT (Table 3).

### 3.4. Resting Energy Expenditure 

**Figure 3:**
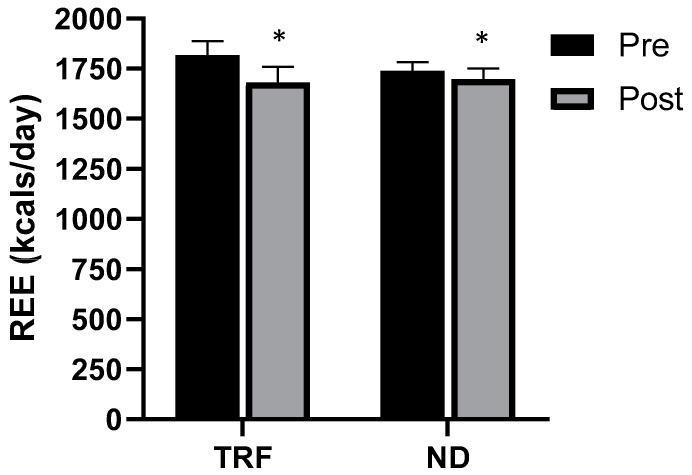
Resting energy expenditure (REE) pre and post four week intervention for both the time-restricted feeding (TRF) and normal diet (ND) groups. Error bars reflect SEM. * = main effect for time.

Main effects for time were noted for REE (*p* = 0.002; η_p_^2^ = 0.324), however, no group × time interactions were observed (Figure 3). 

### 3.5. Muscular Performance: Vertical Jump

No group × time interactions were seen for VJ*_HT_*, VJ*_PP_*, or VJ*_F_*; however, main effects for time were noted for VJ*_HT_* (*p* = 0.002; η_p_^2^ = 0.340; TRF pre: 30.5 ± 6.4 cm, TRF post: 33.9 ± 5.1 cm; ND pre: 34.3 ± 9.4 cm, ND post: 37.7 ± 10.9 cm), and VJ*_PP_* (*p* = 0.018; η_p_^2^ = 0.213; TRF pre: 4007.8 ± 691.5 W, TRF post: 4222.2 ± 518 W; ND pre: 4378 ± 931.8 W, ND post: 4530.8 ± 809.3 W). 

### 3.6. Muscular Performance: Strength and Endurance

**Figure 4:**
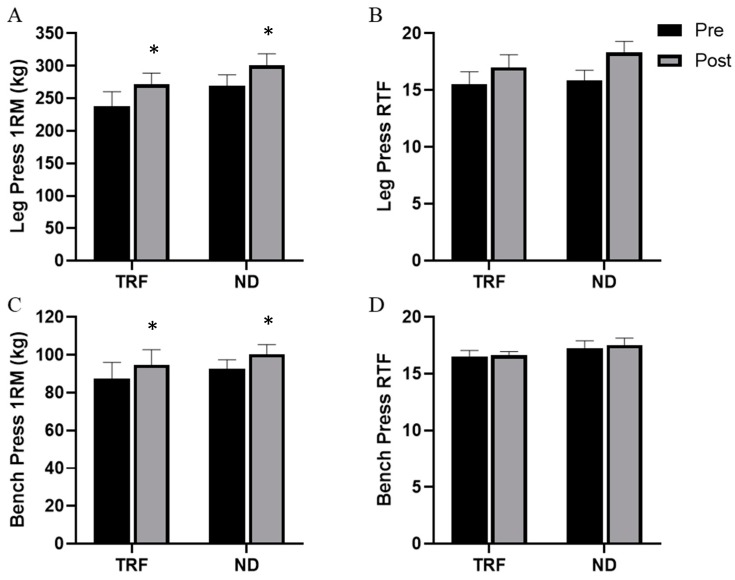
Leg press 1RM (**A**), leg press repetitions to failure (**B**), bench press 1RM (**C**), and bench press repetitions to failure (**D**) over the course of 4-weeks of the intervention from pre to post for both the time-restricted feeding (TRF) and normal diet (ND) groups. Error bars reflect SEM. * = main effect for time.

Main effects for time were noted for LP*_1RM_* (*p* < 0.001; η_p_^2^ = 0.646) and BP*_1RM_* (*p* < 0.001; η_p_^2^ = 0.784), although no group × time interactions were seen for either variable (Figure 4). Furthermore, no main effects for time or group × time interactions were noted for either LP*_RTF_* or BP*_RTF_*.

### 3.7. Psychometric Parameters

Main effects for time were seen for cognitive restraint (*p* = 0.047; ηp2 = 0.155; TRF pre: 38.5 ± 19.5, TRF post: 47.9 ± 16.8; ND pre: 44.1 ± 14.3, ND post: 44.5 ± 17.7). No main effects for time or group × time interactions were noted for emotional eating, uncontrolled eating, perceived recovery between training sessions, VAS (energy, desire to eat, fullness, hunger, and motivation to do physical tasks), or perceptions of daily life stressors.

### 3.8. Blood Biomarkers

A main effect for time was observed for testosterone, leptin, and adiponectin, indicating a decrease in all participants combined. However, no significant group × time interactions were noted. A group × time interaction was noted for cortisol. Post hoc independent samples *t*-tests revealed no differences in cortisol concentrations between ND and TRF at baseline (*p* = 0.652), but higher concentrations in ND as compared to TRF at post (*p* = 0.018). The inter-assay coefficient of variations (CV) and values for all biomarkers can be seen in Table 4.

## 4. Discussion

Recently, fasting regimens such as TRF have gained considerable popularity despite limited empirical support for their advantages when compared to traditional energy-restricted diets. The present study demonstrated short-term TRF did not elicit more favorable alterations in body composition when compared to a normal meal distribution in isocaloric and isonitrogenous conditions. However, our data also suggest that 16/8 TRF does not negatively influence the ability to maintain FFM over four weeks in a hypocaloric state when elevated dietary protein intake and a strenuous resistance training program are present. Additionally, the implementation of daily 16-h fasting periods did not compromise improvements in muscular strength or power over the course of the intervention. 

While the current study was shorter than the three previous TRF and resistance training interventions (i.e., four as opposed to 8-weeks [21,22,23]), relatively similar body composition changes were still noted. Tinsley and colleagues [23] did note apparent differences in LST changes between fasting and non-fasting groups over the course of eight weeks, which, although not statistically significant, may have practical implications for those performing resistance training for body composition enhancement. The researchers postulated that these disparities might be attributed to the differing average daily protein intakes between the groups (ND: 1.4 g/kg/day; TRF: 1.0 g/kg/day). However, despite the lower protein intake, LST was maintained with TRF, most likely due to the resistance training stimulus. In an attempt to eliminate discrepancies in protein intake, Moro and colleagues [21] and Tinsley at al. (2019) [22] matched protein between groups at 1.9 g/kg/day and 1.6 g/kg/day, respectively. Following 8-weeks of 16/8 TRF in resistance-trained males, Moro et al. reported FFM was maintained, lending credence to Tinsley et al.’s (2016) reported hypothesis that protein intake was the likely source of the possible discrepancies in lean mass changes in their previous investigation. Additionally, Tinsley et al. (2019) reported increases in FFM regardless of group (i.e., TRF, TRF plus HMB, control), despite differences in meal timing. Subsequently, the present study similarly supports the three previous interventions, with no differences in the maintenance of FFM between groups, despite differences in meal timing and the resultant discrepancies in daily energy and protein distribution. With the implementation of a 25% caloric deficit, significant decreases in BM attributed to loss of both FM and FFM would be expected in the absence of a structured exercise program and adequate protein intake. As both groups similarly decreased BM, FM, and BF% following the 4-week intervention, and FFM did not change, it can be inferred that the exercise and nutrition intervention was effective for promoting loss of body fat without concurrent lean mass loss. Previous research suggests that a protein intake of ≥2 g/kg/day may be required to maintain FFM during an energy-restricted diet in active individuals [38,39,40] as protein is critical for upregulating muscle protein synthesis, the driving force behind FFM adaptive responses to chronic resistance exercise. While our participants were not consuming 2 g/kg/day, they indeed consumed ~1.8 g/kg/d, a quantity surpassing the 1.6 g/kg/d threshold highlighted by Morton and colleagues [41] for increasing muscle mass when engaging in resistance training as well as in the suggested range of 1.4–2.0 g/kg/day recommended by the International Society of Sports Nutrition [37]. It is plausible the three day per week, full body resistance exercise program, along with a higher protein diet, contributed to these results.

REE decreased similarly for both groups following the 4-week intervention, however, this is in line with previous findings. A systematic review of 90 investigations by Schwartz and Doucet [42] demonstrated an average of 15.4 kcals/kg of weight loss lowering of REE. Interestingly, the average decrement was greater in interventions lasting two to six weeks. The slowing of metabolic rate with weight loss is often attributed to reductions in FFM. However, as our participants maintained FFM, this is not likely to be a contributing factor to the reduction in REE observed in the present investigation.

One of the major discrepancies between the current findings and Moro et al. is that of reductions in FM with the implementation of TRF. Unlike Moro et al. [21], this study demonstrated that when daily caloric intake was equated, the alterations in meal frequency did not play a role in the reductions of FM or BF%. These findings confirmed our hypothesis that overall caloric balance would be the driving factor in changes in body composition, despite alterations in meal timing. However, the differing durations and participant characteristics, particularly training status, between these two studies are noteworthy. Additionally, as both investigations employed self-reported dietary assessments, the reported nutritional intakes should be viewed cautiously.

Participants in the present study increased BP*_1RM_* and LP*_1RM_* to a significant degree, although improvements did not differ between diets. While participants did not increase whole body FFM to a significant degree, results from muscle ultrasonography did demonstrate significant increases in VL and BB CSA. It is plausible that the improvement in both lower body and upper body strength measures over a short period of time can be partially attributed to increases in CSA [43]. While the CSA increases are notable, previous reports highlight that a strong relationship between increases in CSA and muscular strength are not well established until elite levels of training status and proficiency have been achieved [44,45,46]. Given the short training duration in the present investigation, it is more likely that substantial neural adaptations were stimulated in response to the intense resistance training program implemented in recreationally trained participants, and that these adaptations may have primarily contributed to the observed strength increases [47,48,49]. Nevertheless, the increases in muscular strength were similar to outcomes reported by the previous studies in this area [21,23]. While muscular strength improved, muscular endurance did not improve for either group in our investigation. This is unlike Tinsley and colleagues [23], who reported muscular endurance improvements in the lower body. The lack of improvements in muscular endurance in our investigation are likely due to the style of training, rather than the dietary strategies involved.

Another contrary finding from the previous investigation [21] was in regard to the biomarkers serum testosterone and plasma cortisol. Moro and colleagues [21] reported decreases in testosterone in the TRF group over the course of 8-weeks. Conversely, we found no significant differences between groups at any point throughout the investigation with regard to serum testosterone. While the short time period of the current study (4-weeks) may have played a role in these findings, previous investigations have found detectible changes in as little as one to two weeks of resistance training [50,51]. It is worth noting that while we did detect a main effect for time with regard to decreases in testosterone, the actual physiological relevance of observed decreases is highly questionable as serum testosterone levels have been shown to fluctuate as much as 43% throughout a 24 h period [52]. Furthermore, shifts in sleep patterns may disrupt serum testosterone levels by as much as 57 ng/dl [53,54]. Thus, the minor alterations observed are likely not physiologically relevant, despite statistical significance, and should not be over-interpreted. 

Additionally, our findings demonstrated a rise in plasma cortisol only in the ND group over the course of 4-weeks. Rises in cortisol have been shown to occur during hypocaloric periods [55,56,57,58], with greater increases found when participants were asked to closely monitor their diets [55,57,59]. Additionally, fasting has demonstrated an ability to alter the normal circadian rhythm of cortisol rises and falls [60]. While speculative, the period of TRF may have altered the normal spike seen at the time of day when cortisol was assessed. While cortisol assessments were taken at the same approximate time pre- and post-intervention (±2 h), time from the waking hour was not quantified, which may have also affected the measurement [61]. However, the previous investigation of Tinsley et al. indicated no alteration of the cortisol awakening response or changes in average cortisol concentrations with eight weeks of TRF plus resistance training in females [22]. Future investigations should explore the implications of TRF on cortisol levels and rhythms further. Additionally, the discrepancies between the present study’s findings and that of Moro and colleagues (i.e., no change in cortisol) [21] might be attributable to the implementation of a weight-maintenance diet, rather than an energy-restricted diet. 

One of the novel findings of the present investigation was the lack of impact of TRF on perceived athlete readiness. Despite the length of daily calorie abstinence, perceived recovery between training sessions and perceived readiness did not differ between diets. This aspect has not been previously addressed with regard to TRF in resistance training populations, and may be useful when deciding to implement TRF in actively training individuals. Furthermore, the dietary strategy did not significantly impact feelings of energy, motivation to perform physical tasks, fullness, desire to eat, or hunger. However, it is important to highlight that the timing of the surveys may have impacted these findings as they were completed prior to the participants’ workouts, which fell within their feeding window. 

There were a number of limitations to the current study that should not be overlooked when interpreting the findings. First, the use of self-reported dietary intake and basing our dietary analysis on a total of 12 days throughout the 4 weeks are limitations. However, efforts were made by the research team to ensure participants were adhering to all dietary guidelines including interviewing participants during each workout (i.e., three times per week) and reviewing dietary logs at the beginning of each week. We also recognize that the 4-week duration of the study is a limitation and more research needs to be conducted that implements longer timeframes (e.g., 8–24 weeks). Furthermore, our participants’ circadian schedules, sleep schedules, and work/academic schedules varied, which may also influence our findings, especially hormone assessments. Additionally, we only examined recreationally active men. Future investigations should explore diverse populations and various degrees of training status including applications to sport specific performance. Moreover, variants of TRF structure should be explored such as employing TRF on weekdays with returns to normal meal patterns on weekends. Furthermore, future investigations should examine the impact of workout timing (i.e., training performed during or outside the feeding window) on potential performance and body composition outcomes in conjunction with TRF.

## 5. Conclusions

In conclusion, our findings suggest that adopting a TRF style of eating does not enhance reductions in FM over caloric restriction alone during a 4-week hypocaloric diet. Furthermore, daily 16-h fasts do not impact the ability to retain lean mass and improve muscular strength over the course of four weeks in recreationally active populations who resistance train. This dietary regime may be successfully adopted during phases of training in which the goal is to maintain lean mass or muscular strength, while emphasizing body composition improvements.

## Figures and Tables

**Table 1:** Comparison of pretesting participant characteristics.

Characteristic	TRF (*n* = 13)	ND (*n* = 13)	*p*-Value
**Age (yr)**	22.9 ± 3.6	22.5 ± 2.2	0.743
**Height (cm)**	178.1 ± 7.3	177.5 ± 8.8	0.862
**Body Mass (kg)**	82.0 ± 10.6	83.3 ± 15.0	0.804
**Body Fat (%)**	19.9 ± 8.3	18.9 ± 7.4	0.742
**Fat Mass (kg)**	16.8 ± 8.6	16.3 ± 8.5	0.886
**Fat Free Mass (kg)**	65.2 ± 6.6	67.0 ± 9.5	0.588
**Vertical Jump Height (cm)**	30.5 ± 6.4	34.2 ± 9.4	0.241
**Bench Press 1RM (kg)**	87.6 ± 30.4	92.6 ± 17.2	0.606
**Leg Press 1RM (kg)**	238.1 ± 80.0	269.5 ± 60.8	0.271
**Bench Press RTF**	16.5 ± 1.9	17.2 ± 2.4	0.421
**Leg Press RTF**	15.5 ± 3.9	15.8 ± 3.3	0.830

yr = years; cm = centimeters; kg = kilograms; 1RM = 1 repetition maximum; RTF = repetitions till failure; TRF = time restricted feeding; ND = normal daily feeding.

**Table 2:** Macronutrient and calorie intake averages across the four week investigation (two weekdays and one weekend day per week was collected).

Dietary Component	TRF (*n* = 13)	ND (*n* = 13)	*p*-Value
**Protein (g/kg)**	1.83 ± 0.10	1.83 ± 0.07	0.956
**Carbohydrate (g/kg)**	2.28 ± 0.71	2.25 ± 0.51	0.889
**Fat (g/kg)**	0.84 ± 0.26	0.85 ±0.22	0.917
**Total Kcal**	1946 ± 310	1939 ± 260	0.950
**Relative Kcal (g/kg)**	23.81 ± 3.25	23.64 ± 3.07	0.892

Results are presented as mean ± SD; g/kg = grams per kilogram of body mass; TRF = time restricted feeding; ND = normal daily feeding.

**Table 3:** Muscle morphology for the vastus lateralis, rectus femoris, and bicep brachii at pre and post four week intervention.

Muscle	TRF Pre	TRF Post	ND Pre	ND Post	*p*-Value	η_p_^2^
**VL CSA (cm** ^**2**^ **)**	31.0 ± 7.8	33.7 ± 5.3	36.0 ± 7.3	37.5 ± 5.3	0.002*	0.329
**VL MT (cm)**	1.8 ± 0.4	2.0 ± 0.4	2.1 ± 0.4	2.2 ± 0.4	0.065	0.135
**VL EI (AU)**	57.4 ± 12.8	53.1 ± 12.4	49.2 ± 8.1	46.6 ± 7.4	0.001*	0.368
**RF CSA (cm** ^**2**^ **)**	12.6 ± 2.4	12.7 ± 2.1	14.6 ± 4.3	15.0 ± 4.5	0.228	0.060
**RF MT (cm)**	2.39 ± 0.4	2.35 ± 0.4	2.71 ± 0.5	2.70 ± 0.5	0.462	0.023
**RF EI (AU)**	63.8 ± 16.7	63.8 ± 18.2	58.2 ± 12	55.3 ± 13.8	0.182	0.073
**BB CSA (cm** ^**2**^ **)**	11.9 ± 2.2	12.7 ± 1.9	12.6 ± 2.1	13.3 ± 2.5	0.013*	0.229
**BB MT (cm)**	2.3 ± 0.4	2.6 ± 0.2	2.3 ± 0.4	2.6 ± 0.3	<0.001*	0.534
**BB EI (AU)**	93.3 ± 12.4	88.1 ± 11.9	88.2 ± 13.6	87.9 ± 7.3	0.177	0.074

Results are presented as mean ± SD; VL = Vastus Lateralis; RF = Rectus Femoris; BB = Bicep Brachii; CSA = Cross Sectional Area; MT = Muscle Thickness; EI = Echo Intensity; cm = centimeters; AU = Arbitrary Units; * signifies *p* < 0.05 for main effect for time.

**Table 4:** Changes in blood biomarkers.

Biomarker	TRF Pre	TRF Post	Inter-Assay CV (%)	ND Pre	ND Post	Inter-Assay CV (%)	*p*-Value (Main Effect for Time)	η_p_^2^ (Main Effect for Time)	*p*-Value (Group × Time Interaction)	η_p_^2^ (Group × Time Interaction)
**Serum Testosterone (ng/dL)**	587.8 ± 8.7	581.2 ± 8.7	1.3	557.2 ± 8.7	556.1 ± 10.3	1.1	0.041 *	0.163	0.140	0.088
**Plasma Cortisol (ng/mL)**	118.3 ± 31.9	106.1 ± 49.7	6.6	119.2 ± 37.7	150.7 ± 39.1	4.3	0.348	0.037	0.040 *^†^	0.165
**Serum Adiponectin (µg/mL)**	5.5 ± 2.3	4.7 ± 1.8	3.2	5.1 ± 2.0	4.4 ± 1.9	4.7	<0.001 *	0.417	0.721	0.005
**Plasma Leptin (pg/mL)**	4680 ± 4566	3689 ± 4512	4.2	4773 ± 3884	3629 ± 3105	5.6	0.001 *	0.351	0.800	0.003
**Plasma Ghrelin (pg/mL)**	2226 ± 1205	2296 ± 945	1.2	3107 ± 1172	3217 ± 1216	2.8	0.427	0.027	0.856	0.001

Results are presented as mean ± SD; * denotes *p* < 0.05; ^†^ signifies a post hoc independent samples *t*-test revealing significant differences at post presented in the text.; TRF = time restricted feeding; ND = normal daily feeding; CV = coefficient of variation.
